# Machine Learning and BMI Improve the Prognostic Value of GAP Index in Treated IPF Patients

**DOI:** 10.3390/bioengineering10020251

**Published:** 2023-02-14

**Authors:** Donato Lacedonia, Cosimo Carlo De Pace, Gaetano Rea, Ludovica Capitelli, Crescenzio Gallo, Giulia Scioscia, Pasquale Tondo, Marialuisa Bocchino

**Affiliations:** 1 Department of Medical and Surgical Sciences, University of Foggia, 71121 Foggia, Italy; 2 Department of Radiology, Monaldi Hospital, AO dei Colli, 80131 Naples, Italy; 3 Respiratory Medicine Unit, Department of Clinical Medicine and Surgery, Federico II University of Naples, 80131 Naples, Italy; 4 Department of Clinical and Experimental Medicine, University of Foggia, 71121 Foggia, Italy

**Keywords:** idiopathic pulmonary fibrosis, GAP index, machine learning, mortality, body mass index, nintedanib, pirfenidone

## Abstract

Patients affected by idiopathic pulmonary fibrosis (IPF) have a high mortality rate in the first 2–5 years from diagnosis. It is therefore necessary to identify a prognostic indicator that can guide the care process. The Gender-Age-Physiology (GAP) index and staging system is an easy-to-calculate prediction tool, widely validated, and largely used in clinical practice to estimate the risk of mortality of IPF patients at 1–3 years. In our study, we analyzed the GAP index through machine learning to assess any improvement in its predictive power in a large cohort of IPF patients treated either with pirfenidone or nintedanib. In addition, we evaluated this event through the integration of additional parameters. As previously reported by Y. Suzuki et al., our data show that inclusion of body mass index (BMI) is the best strategy to reinforce the GAP performance in IPF patients under treatment with currently available anti-fibrotic drugs.

## 1. Introduction

Idiopathic pulmonary fibrosis (IPF) [1,2] is a rapidly progressive interstitial lung disease of unknown cause, which usually affects men over 65 years of age with a history of smoking. Diagnosis of IPF is often complex and based on many specialists’ experience, not only pulmonologists but also radiologists and histopathologists. High-resolution computed tomography (HRCT) findings, especially after the update in the international guidelines, are crucial in the diagnostic approach, and in case of a radiological usual interstitial pneumonia (UIP) pattern, but also likely in a probable UIP pattern, the diagnosis of idiopathic pulmonary fibrosis is made. A multidisciplinary approach has become more and more relevant, especially in uncertain radiological and clinical conditions and in the absence of histopathologic samples, which requires a multi-specialist presence as evidenced by the newest published guidelines [3].

Symptoms are commonly a dry cough, progressive dyspnea, fatigue, and a progressive decline in capability and independence during daily activities. The most characteristic sign identifiable during the physical examination of the patient is the “velcro-type” crackle during auscultation due to traction bronchiectasis but also, in the advanced stage of the disease, the clubbing of the fingernails due to chronic hypoxemia.

Idiopathic pulmonary fibrosis is associated with a poor prognosis, with high mortality rates in the first 2–5 years from diagnosis in untreated cases. Pharmacological treatment is based exclusively on two approved drugs, which are unfortunately useful only in slowing the evolution of the disease. In 2011, pirfenidone was the first treatment approved [4,5] for idiopathic pulmonary fibrosis for its favorable benefit-risk ratio due to trial results showing a decrease in disease progression in lung function, exercise tolerance, and improvement in survival compared to a placebo. It is an orally bioavailable antifibrotic agent and has also anti-inflammatory and antioxidant effects. After four years, nintedanib was approved [6,7], the second and, currently, the last pharmacological treatment. It is an intracellular inhibitor of tyrosine kinase that causes an inhibitory effect on fibroblast proliferation and differentiation, delivering significant effects in reducing lung function decline. Adverse effects are possible after the administration of both drugs. Pirfenidone is more often associated with photosensitivity and skin rash while nintedanib is most frequently associated with diarrhea. The two antifibrotic drugs are also associated with liver toxicity, which is why treated patients need to have blood samples taken for transaminase control.

Many trials are in progress to develop newer and more effective drugs to not only slow but also stop the degeneration caused by fibrosis, but until now, unfortunately, there are no effective treatments, which is why the evaluation of the prognosis remains an actual and debated question, as we can also see from the continuous research into more complex biomarkers [8].

Psychological assistance and rehabilitation are central in the treatment of patients affected by IPF, not only due to the poor prognosis but also due to the constant and progressive worsening of the quality of life.

Depression and anxiety are indeed very common in patients affected by IPF [9], regardless of survival or hospitalization, highlighting the need for better detection and treatment of this condition.

An interesting finding is also that patients affected by idiopathic pulmonary fibrosis are often associated with gastro-esophageal reflux, which can cause a worsening of the disease but also several other respiratory diseases such as laryngeal disorders, sinusitis, and chronic coughing [10]. The most frequently used drugs to treat gastro-esophageal reflux are proton pump inhibitors, which seem to be related to a worsening of the psychological status of treated patients [11].

Furthermore, respiratory rehabilitation is a fundamental part of the treatment of IPF, which is useful both to slow the decline of respiratory function and to improve the quality of life [12].

A mention must be made, given the pandemic situation and due to their frail clinical condition, of the importance of early treatment of patients affected by IPF that react positively to the nasopharyngeal swab for research into SARS-CoV-2 [13] and the importance of the creation of adequate paths to post-covid rehabilitation [14] and follow-up [15].

In selected patients, a viable treatment option is lung transplantation [16], which can be unilateral or bilateral, although a recent study [17] has demonstrated no survival advantage with bilateral transplantations. Moreover, unilateral transplantations have reduced wait time, are easier to perform, and it is possible to improve the health of two patients from one donor.

Nowadays the most used prognostic indexes for IPF are the GAP [18] and TORVAN [19] indexes. Developed in 2012, the composite GAP index relies on the assessment of gender (G), age (A), and physiology (P), the latter including two conventional lung function parameters: the forced vital capacity (FVC) and the diffusion lung capacity for CO (DLCO). It assigns a value to each variable (gender: female = 0 vs. male = 1; age: ≤60 years = 0, 61–65 years = 1, >65 years = 2; % predicted FVC: >75% = 0, 50–75% = 1, <50% = 2; % predicted DLCO: >55% = 0, 36–55% = 1, ≤35% = 2, cannot perform = 3). The final score resulting from the sum of the addenda allows the estimation of the average risk of mortality at 1, 2, and 3 years by disease stage (i.e., score 0–3 = stage I; score 4–5 = stage II; score 6–8 = stage III). First validated in untreated IPF patients, newer publications are evaluating GAP in treated ones [20].

Nevertheless, over the last ten years, the knowledge and treatment of idiopathic pulmonary fibrosis have evolved, and the search for more effective and personalized tools has become a priority to improve the quality of patient care. The prognosis of IPF patients indeed depends on many variable comorbidities, and clinical, psychological, and physical conditions [21,22,23,24,25].

The TORVAN index was developed in 2019 by Torrisi et al. [19] with the purpose of integrating age, FVC, and DLCO, and also the evaluation of comorbidities, such as systemic hypertension, atrial arrhythmias, diabetes mellitus, major depressive disorder, gastroesophageal reflux, pulmonary hypertension, lung cancer, and valvular heart disease to estimate the TORVAN stage related to the prognosis. 

Torrisi et al. did not include gender in their index because they evaluated that sex becomes a less important parameter when considered in relation to comorbidities. In their study, the most important comorbidities resulted from gastroesophageal reflux, pulmonary hypertension, lung cancer, valvular heart disease, and atrial arrhythmias. 

The GAP index, however, still remains the most widely used prognostic tool in daily clinical practice, which is why we decided to focus on it, leaving the possibility of a further machine learning (ML) approach to TORVAN.

Recently, Y. Suzuki et al. [26] have shown that the combination of the GAP index with the body mass index (BMI) successfully identified four sub-groups of IPF patients with different prognoses before the initiation of anti-fibrotic therapies. 

Machine learning is a branch of artificial intelligence (AI) that focuses on the use of data and algorithms to imitate the way humans learn, automatically improving from experience without being explicitly programmed. ML displays powerful problem-solving capabilities along with the ability to detect the interaction between numerous variables, thus allowing the development of prediction models. In light of this potential, although burdened by some limitations, the application of machine learning has been explored in clinical settings with encouraging results [27]. 

Given these considerations, the purpose of our study was to validate the GAP index with an ML approach in a retrospective cohort of IPF patients treated in a real-life setting with the two currently available anti-fibrotic drugs, pirfenidone and nintedanib. 

As an additional aim, we tested the possibility of integrating the GAP with other clinical variables to improve its prognostic performance.

## 2. Machine Learning Operation

Artificial intelligence enables, through machine learning, tasks that are too difficult to solve with fixed programs written and designed in a traditional way. From a scientific and philosophical point of view, ML is interesting because the development of its understanding promotes understanding of the principles underlying intelligence.

Machine learning is achieved through the application of special learning mechanisms (algorithms); in particular, an ML algorithm is an algorithm that is able to learn from data [28] through the exploration of correctly predicted data examples that can instruct the algorithm to build an effective predictive model. In order to obtain the best choice of the dataset’s variables (attributes) to employ in the predictive model, a ranking of the attributes is performed, which scores them according to their correlation with the value (class) to be predicted.

Feature scoring and ranking can help in understanding the data in supervised settings. This is typically achieved by means of entropy-based [29,30,31] or correlation-based [32,33,34] indicators.

## 3. Materials and Methods

We retrospectively collected the data of 211 patients affected by idiopathic pulmonary fibrosis attending two referral interstitial lung disease (ILD) centers (Universities of Foggia and Federico II of Naples) at the time of first diagnosis. 

Variables collected at baseline (before the initiation of anti-fibrotic therapies) included age (69.5 ± 7.5 years), gender (males = 186; females = 61), BMI (29.5 ± 4.4 kg/m^2^), smoking habit (active or former smokers = 166), type of IPF diagnosis (based on chest imaging = 175; based on lung histology = 36), and disease phenotype (sporadic = 181; familial = 30). Additional study parameters were those related to the lung function, such as FVC (77 ± 19% of predicted), forced expiratory volume/1st second (FEV1) (83 ± 20% of predicted), total lung capacity (TLC) (62 ± 16% of predicted), DLCO (50 ± 14% of predicted), and the initial and final O2 peripheral saturation (SpO_2_) recorded at the 6 min walk test (6MWT) (95 ± 2% and 87 ± 6%, respectively), along with the distance walked (367 ± 133 m). The GAP scoring was collected as well (3.8 ± 1.4).

The open-source machine data program Orange (https://orange.biolab.si/ (URL accessed on 06 June 2022) was used to design the machine learning approach to validate the GAP index in estimating the 3 year mortality rate in our study cohort. 

ML allows us to analyze large amounts of data with the advantage of detecting possible interactions among many different variables, but it is still a too complex predictive model, which is why its use cannot be routine.

The application of ML to our work started with the selection of the most relevant features to be used in the predictive models, which was carried out by means of principal component analysis (PCA) applied to the patient dataset. Subsequent processing was carried out by “feature ranking” in order to further reduce the number of features in the final model. We used the FCBF (fast correlation–based filter) method—an entropy-based measure, which also identifies redundancy due to pairwise correlations between features—choosing the features with a ranking value greater than zero.

Finally, we trained and tested a series of classification models in order to verify our approach to the GAP index predictivity enforcement by means of BMI. In particular, we built three predictive models using the following ML methods:

- Logistic regression (a classification algorithm with LASSO regularization)
- Naive Bayes (a fast and simple probabilistic classifier based on Bayes’ theorem with the assumption of feature independence)
- Neural network (a multi-layer perceptron algorithm with backpropagation)

The learning methods showed slightly different classifier performance measures (such as classification accuracy (CA) and AUC (area under the ROC curve)), which we used for validating their performance by means of cross-validation (a sampling method that allows the reduction of overfitting problems), confusion matrix (which shows proportions between the predicted and actual class) and ROC analysis (which plots a true positive rate against a false positive rate of a test, giving a robust assessment of the predictive model).

These validation tools led to approximately the same results in terms of accuracy and F1 score (0.71), with an AUC (area under ROC curve) of 0.74, confirming the soundness of our hypothesis.

A detailed description of the methodology employed to perform the analysis carried out in this work is reported in a previously published paper by C. Mencar et al. [35].

The study was approved by the Ethics Committee of the Azienda Ospedaliera dei Colli, Naples, Italy. Data of interest for the analysis were anonymously collected into a dedicated database. Informed consent was not required due to the retrospective nature of the study.

## 4. Results

The overall 3 year mortality rate in our study cohort of IPF patients treated with pirfenidone or nintedanib was 36.7% (78 patients have died). This finding was aligned with the mortality prediction based on the GAP index at baseline (Table 1). No differences were observed in the two treatment arms.

Table 2 shows the accuracy and other ML-derived parameters calculated by means of different methods when evaluating the sole GAP index. Not dissimilar data were obtained by considering the single GAP components (gender, age, FVC, and DLCO) taken individually, suggesting that machine learning did not produce a further improvement. The best result obtained was for logistic regression (AUC = 0.72; CA = 0.69; F1 = 0.67).

We considered other variables not included in the index such as 6MWT parameters/distance, initial and final SpO_2_, type of diagnosis (radiological or histological), type of therapy, TLC%, and Torvan Index. The best results were obtained by considering the final peripheral saturation of oxygen, the type of diagnosis, and type of therapy (Logistic regression: AUC 0.70–F1 0.66, Neural Network: AUC 0.70–F1 0.65).

A clear improvement in the results was obtained by integrating the analysis of the individual GAP parameters with the value of BMI (Table 3).

## 5. Discussion

In the present work, we have explored the possibility of improving the prognostic value of the GAP index in idiopathic pulmonary fibrosis patients treated with anti-fibrotics by means of machine learning algorithms employing optimal predictive variables. In order to do this, we measured the performance of various additional parameters from the available dataset through entropy and correlation indicators, which led to the choice of body mass index as the best additional parameter to add to the predicting model.

Although this result may not seem to be of relevant novelty, it highlights the importance of the “machine learning” approach in the prognostic evaluation of diseases (in our case of IPF) for supporting the diagnostic ability of physicians in the execution of their clinical activities which, if otherwise performed “by hand”, would often lead to unsatisfactory results.

The machine learning approach was utilized before in relation to idiopathic pulmonary fibrosis, both for diagnosis and prognosis.

Already in 2011, Flietstra B. et al. [36] used ML to automate the analysis of crackles to distinguish them among IPF, congestive heart failure (CHF), and pneumonia. They analyzed distinctive features of IPF crackles separating them from those in patients with pneumonia or CHF, potentially aiming to avoid eventual medication errors and to ease diagnosis.

Furukawa T. et al. [37] tried to utilize ML to detect IPF in a group of ILD patients and to differentiate them. They used a combination of deep learning, used for semantic segmentation of HRCT images, and an ML algorithm to retrospectively analyze HRCT images of 1068 patients affected by interstitial lung disease and, without data from non-invasive examinations, they reached the target to identify patients affected by IPF in a population with various ILDs. 

Pan J. et al. [38] recently published that machine learning may be used to identify novel radiological disease progression in lung CT of IPF patients. In their work, they proceed to answer three main questions.

With respect to the first question, whether it is possible to identify newer lung pattern types associated with radiological disease progression, they affirm this possibility is feasible.

The second question is if patients with different radiological disease progression have different future outcomes, but in this case, the answer is more difficult. They actually found significantly different outcomes, but by extending the evaluation to an external cohort with different scanner manufacturers, they only observed a comparable trend without reaching significance.

Lastly, with respect to the possibility of visualizing the transition pathways of components that correspond to frequently occurring changes in lungs during radiological progression, they specify the analysis shows that is possible to extract these transition networks from patients through follow-up examinations.

This is an important study because they obtained a substantial comparable performance between ML and expert radiologists, even if in a small cohort of patients, opening the way to further informatics improvement also in the radiological prediction of prognosis.

To the best of our knowledge, this is the first attempt to assess whether the application of machine learning can improve the prediction accuracy of the Gender-Age-Physiology index in IPF patients treated with the anti-fibrotic drugs, pirfenidone and nintedanib. 

Our results show that ML did not further improve the GAP performance; this observation was such both when considering the GAP as a composite index and when evaluating individually its single components.

We read the validation of the GAP index based on the comparison between mortality in the Korean population [39] and the population selected in the original article by Ley. In this article from 2015, there was reliability in the survival estimation at 1 year, with worsening of the results in the estimations at 2 and 3 years, with lower mortality than predicted, even though these data were limited by differences in the cohorts (better value of FVC and DLCO, lower age). Data in our analysis show higher mortality in the first and second stage at 3 years and, on the contrary, lower mortality in the third stage, especially because all our patients were treated with antifibrotics (no statistical differences were highlighted between pirfenidone and nintedanib in the analysis) while in the article by Ley, no therapy was approved yet, which is why we expected an improvement in the patients’ survival in all stages.

This result seems to corroborate the thesis presented by S. Suissa and K. Suissa in their very recent article. They affirm that in many studies, the reduction in mortality after antifibrotic treatment with pirfenidone or nintedanib is a result of immortal time bias that exaggerates the benefits of drugs, even though further studies are requested [40].

During our analysis, we also tried to find a more accurate index as an alternative one in cases where we did not have the data needed to calculate GAP.

As already cited, the best results were obtained with the final peripheral saturation of oxygen, the type of diagnosis, and the type of therapy, but these parameters and machine learning results did not encourage us to proceed in this research, leaving the possibility of pursuing this approach in future studies.

A mild relation is visible in the approach to improving the GAP index attempted by Chandel A. et al. [41], where they integrated into GAP parameters 6MWD and exertional hypoxia.

They propose a Distance-Oxygen-GAP (DO-GAP) in which they suggest that 6MWD is a more reproducible measure of the disease that improves GAP because it assesses overall functional ability and evaluates also the impact of other comorbidities (such as cardiovascular, rheumatologic, or neurologic impact). This is a very interesting result; unfortunately in our ML approach, with our dataset of patients, we obtained only a mild and not significant improvement.

In addition, we found it really interesting to further classify, as proposed in the article by Suzuki et al., patients considering BMI. In the paper, it is proposed to move to the next stage patients that have a BMI lower than the cut-off (24 kg/m^2^), an approach that caused a reclassification, especially of patients with GAP stage II (approximately 85% of their cohort). This theory seems confirmed by our validation through machine learning. Furthermore, as cited in the article, a faster decline in FVC among patients with lower BMI and weight loss > 5% during 52 weeks was also reported in the second analysis of the INPULSIS study.

There are some limitations in this study. We used ML analysis only to evaluate the GAP index. While the TORVAN index certainly allows the inclusion, in the estimation of prognosis, also the presence of comorbidities, as already cited, we preferred to focus on the GAP index because is still the most widely used, leaving the possibility of evaluating TORVAN in a further analysis.

The GAP index was published and tested in an untreated population, so its absence could be another limitation; however, all patients in our dataset are treated with antifibrotics according to guidelines.

Lastly, the dataset is composed of a population geographically limited to Southern Italy; nevertheless, the analyzed patients are from two separate centers and the number of the patient sample is not negligible for a rare disease.

## 6. Conclusions

In conclusion, the analysis of data, based only on our dataset, demonstrates that even if the GAP index utilizes common variables for patients affected by idiopathic pulmonary fibrosis, making it very simple to measure, is still reliable in terms of prognosis, confirmed by the fact that more complicated calculation systems such as machine learning, utilizing only clinical parameters, does not provide better results. On the other hand, the addition of the parameter BMI, as suggested in the paper by Y. Suzuki et al., seems to further improve the predictive accuracy of GAP.

## Figures and Tables

**Table 1:** Comparison between GAP-predicted and observed mortality in our study cohort of IPF patients.

Disease Stage	3 Year Mortality Estimated by GAP	3 Year Observed Mortality
GAP-I	16%	21%
GAP-II	40%	46%
GAP-III	77%	70%

**Table 2:** Validation of the GAP index by means of machine learning AUC = Area Under Curve; CA = Classification Accuracy; F1 = harmonic mean between Precision and Recall.

	AUC	CA	F1
Logistic Regression	0.70	0.71	0.71
Neural Network	0.69	0.71	0.71
Naive Bayes	0.67	0.71	0.71
Stochastic gradient descent	0.68	0.71	0.71
Random Forest	0.68	0.71	0.71

**Table 3:** Validation of the GAP index through the individual analysis of its components (gender, age, FVC, and DLCO) in addition to BMI.

	AUC	CA	F1
Naive Bayes	0.78	0.70	0.70
Logistic Regression	0.77	0.70	0.69
Support Vector Machine	0.74	0.72	0.71
Random Forest	0.73	0.70	0.70
K-Nearest Neighbors	0.71	0.72	0.71

## Data Availability

Data available by contacting the corresponding author.
